# Contracting Skeletal Muscle Releases Antioxidant-Enriched Extracellular Vesicles and Non-vesicular Extracellular Nanoparticles

**DOI:** 10.21203/rs.3.rs-10978006/v1

**Published:** 2026-10-08

**Authors:** Sarah Pribil Pardun, Li Yu, Saadyeh Rashidi, Chittibabu Guda, Lie Gao

**Affiliations:** 1 Department of Anesthesiology, University of Nebraska Medical Center, Omaha, NE 68198, USA; 2 Department of Genetics, Cell Biology, and Anatomy, University of Nebraska Medical Center, Omaha, NE 68198, USA

**Keywords:** extracellular vesicles, non-vesicular extracellular nanoparticles, electrical pulse stimulation, Nrf2 activation, cardioprotection, interorgan crosstalk

## Abstract

While the benefits of exercise are well-documented, the underlying molecular mechanisms remain to be fully elucidated. Previous research in our lab demonstrated that electrical pulse stimulation (EPS) of cultured C2C12 myotubes activates the Nrf2/antioxidant pathway. In the study presented in this manuscript, we hypothesized that these upregulated antioxidant enzymes are secreted into the conditioned medium (CdM) *via* extracellular vesicles (EVs) and non-vesicular extracellular nanoparticles (nvEPs). We also hypothesized that these antioxidant-enriched particles protect H9c2 cardiomyocytes from H_2_O_2_-induced damage. EVs/nvEPs were isolated from the CdM of EPS-treated C2C12 cells and compared to those from non-stimulated (NS) cells using differential ultracentrifugation. Transmission electron microscopy (TEM) revealed that the isolated particles displayed a typical morphology, with EVs showing a cup-shaped structure and lipid bilayer membrane, and nvEPs exhibiting a pill-like and amembranous structure with high electron density. Western blot analysis demonstrated that EVs/nvEPs from EPS-C2C12 myotubes contained higher levels of antioxidant proteins (NQO1 and GSTA2) than those isolated from non-stimulated myotubes. Using a CCK-8 viability assay, we found that H9c2 cells pretreated with C2C12-derived nvEPs showed varying degrees of survival following H_2_O_2_ challenge. Our findings demonstrate that the EVs/nvEPs released from EPS-C2C12 myotubes contain enriched antioxidant enzymes, which protect cardiomyocytes from oxidative stress.

## Introduction

Previously, our lab explored a method of exercise simulation in which C2C12 myotubes were subjected to electrical pulse stimulation (EPS) for one hour per day for four consecutive days. This stimulation resulted in substantially elevated antioxidant enzyme protein expression downstream of the Nrf2 pathway as compared to non-stimulated (NS) control cells. This prior study provides evidence that short-term contraction can protect skeletal myotubes against subsequent oxidative stress-induced cytotoxicity, potentially *via* Nrf2-evoked activation of intracellular antioxidant mechanisms, offering novel insight into SkM redox signaling responses to exercise^1^.

While it is widely understood that the systemic benefits of exercise involve SkM, the precise mechanisms by which contracting SkM contributes to the cardioprotective effects of exercise remain to be fully determined. SkM is increasingly recognized as a secretory organ that releases contraction-sensitive signaling factors capable of communicating with other organs^2, 3^. In the cardiovascular system, exercise and exercise-induced circulating EVs have been shown to protect the heart against ischemia-reperfusion (I/R) injury, but the specific SkM-derived cargo and mechanisms responsible for this protection are still incompletely understood^4, 5^. This study aimed to uncover a mechanism by which SkM during exercise may confer cardioprotection by studying the cargo of EVs and nvEPs released from contracting SkM, such as that which occurs during exercise.

Cells secrete signaling molecules, including proteins, lipids, and nucleic acids as a means of communicating with one another. EVs are membrane-bound particles which are naturally released from cells into the extracellular space and are marked by a lipid bilayer^6-8^. While initially thought to be a normal means for cells to dispose of unwanted material, EVs have become increasingly recognized as important mediators of intercellular communication and facilitators of cargo transfer from one cell to another^9, 10^. EVs are used as packaging for these signaling molecules as a means of avoiding rapid degradation, and they allow for local and long-distance intercellular communication to occur successfully. EVs come in a variety of subpopulations which vary in size, morphology, composition, and intracellular origins^6, 11, 12^. The subpopulations are often classified based on biogenesis and origin. There are three main sources of biogenesis that have been defined in the literature: 1) apoptotic EVs which are generated by the fragmentation of cells undergoing apoptosis, 2) ectosomes which are generated by the direct outward budding of the plasma membrane, and 3) exosomes which are generated by the inward budding of endosomal components; these later fuse with the plasma membrane of the cell before secretion^13^.

Within the last decade, the extracellular particle field has grown to include non-vesicular extracellular nanoparticles (nvEPs). The first of these new nanoparticles, exomeres (EM), were described in 2018 by Zhang et al. using asymmetric flow field-flow fractionation^14, 15^. Three years later, in 2021, Zhang et al. identified a group of even smaller nanoparticles, which they termed supermeres (SM)^16, 17^. nvEPs have distinguishing features which differentiate them from EVs. The most prominent of these features is their lack of a lipid bilayer membrane. Some have an outer shell of a monolayer while others are completely amembranous^18^. These nvEPs are smaller than EVs and are generally less than 50 nm in diameter^14, 15, 17, 19, 20^. They also differ in their type of cargo. While EVs are known to carry proteins, nucleic acids, lipoproteins, and cellular debris, nvEPs, due to their size and non-vesicular structure, primarily carry proteins and nucleic acids^15, 17, 18^ .

Both EVs and nvEPs are known to mediate pathological and physiological processes and to play various roles in disease states. As SkM is the largest organ by mass within the body, it has been suggested that it is a major source of circulating EVs, especially during and after exercise^21-23^. SkM-derived EVs are thought to enter the bloodstream^21^ where they may participate in muscle-organ crosstalk. However, the role of EVs and nvEPs in cardiovascular health is not well understood, particularly with respect to whether contracting SkM can export antioxidant enzymes that are useful to recipient cardiac cells. This question is important because EVs produced under oxidative or adaptive stress conditions can carry antioxidant or redox-related proteins and influence oxidative stress responses in recipient cells^24, 25^. In this study, we aimed to further investigate the role of SkM-derived EVs and nvEPs in cardiovascular health.

Based on previous findings in our lab^1^, we hypothesized that antioxidant enzymes upregulated in contracting C2C12 myotubes are not limited to intracellular defense but are also secreted into the CdM *via* EVs and nvEPs. We further hypothesized that these antioxidant-enriched particles protect H9c2 cells, a rat heart cell line, from hydrogen peroxide (H_2_O_2_)-induced damage. To test this hypothesis, EVs/nvEPs were isolated from CdM of EPS-treated C2C12 myotubes and compared with particles isolated from NS-control myotubes. Particle morphology was examined by transmission electron microscopy (TEM), antioxidant cargo was examined by Western blot analysis of NQO1 and GSTA2, and cardiomyocyte protection was assessed by CCK-8 viability assay following H_2_O_2_ challenge. Together, this design allowed us to test a potential mechanism by which contracting SkM releases antioxidant-enriched EVs/nvEPs that may be taken up by cardiomyocytes to enhance antioxidant defenses and reduce oxidative stress-related cardiac injury, such as the damage that occurs during I/R injury.

## Materials and Methods

### 1. Cell Culture

To minimize animal use in accordance with the 3Rs (Replacement, Reduction, and Refinement), all cell cultures were maintained using Dulbecco’s Modification of Eagle’s Medium (DMEM/HIGH GLUCOSE; HyClone, Logan, UT, USA). Where animal-derived products such as fetal bovine serum (FBS; Gibco/ThermoFisher Scientific New Zealand Limited, Auckland, New Zealand) were necessary, they were sourced from certified ethical vendors complying with international animal welfare regulations. The C2C12 cell line (CRL-1722, ATCC, Manassas, VA, USA) was used in these experiments. Cells were seeded in a 6-well plate at ~50,000 cells per well and grown for one day in complete media (CpM) (Dulbecco’s Modification of Eagle’s Medium (DMEM/HIGH GLUCOSE; HyClone, Logan, UT, USA) supplemented with 10% fetal bovine serum (FBS; Gibco/ThermoFisher Scientific New Zealand Limited, Auckland, New Zealand) and 1% penicillin/streptomycin (P/S)). After ~24 h, the media was changed from CpM to differentiation media (DM) (DMEM supplemented with 5% horse serum (HS; Gibco/ThermoFisher Scientific) and 1% P/S), in which the cells differentiated from myoblasts to myotubes after four days in culture at 37 °C, 5% CO_2_, and 95% humidity. On the fourth day, the media were replaced by fresh DM, and the cells underwent electrical pulse stimulation (EPS)^1^.

### 2. Electrical Pulse Stimulation (EPS) and Cell Collection

An electrical current was delivered to the C2C12 myotubes via stimulation electrodes designed and fabricated in our lab. These electrodes were built on the lids of standard 6-well plates, similar to what has previously been reported^26^. On the first day of EPS, the regular 6-well plate lids were replaced by EPS lids containing the electrodes, which were connected to a single-channel pulse generator (A310 Accupulse, WPI, Sarasota, FL, USA). The EPS lids were cleaned before and after each study using the following protocol: The EPS chamber was flooded with 70% ethanol for 10 min. The electrodes and EPS lids were washed with ~3 mL of sterile PBS. Finally, the EPS lid was exposed to UV light for 10–30 min in the cell-culture laminar flow hood. The C2C12 myotubes underwent EPS at 10 V, 50 Hz, 10 ms, 0.3 s/3 s for 1 h/day for 4 days. At the end of the EPS, the cells were rinsed with 200 μL of sterile PBS and then trypsinized with 200 μL of 0.05% trypsin for 2 min at 37 °C. The cells were then collected and transferred to 1.5 mL EP tubes, which were stored at −80 °C for future analysis^1^.

### 3. EV and nvEP Isolation

The CdM of differentiated C2C12 myotubes either having undergone EPS or not (controls) was collected immediately after EPS. EVs/nvEPs were isolated from this media (Figure 1). Samples were balanced within 0.010 g of one another with PBS and run on a benchtop centrifuge at 500 x g and 4 °C for 10 min. The supernatant was transferred to new 15 mL falcon tubes, balanced with PBS, and centrifuged at 2,000 x g and 4 °C for 20 min. The pellet from the previous centrifugation, containing cells and cell debris, was discarded. Upon completion of the centrifuge run, the supernatant was transferred to new 15 mL falcon tubes, balanced with PBS, and centrifuged at 10,000 x g and 4 °C for 40 min. The pellet from the previous centrifugation, containing cell debris and apoptotic bodies, was discarded. Upon completion of the centrifuge run, the supernatant was transferred to UltraClear ultracentrifuge tubes (Beckman Coulter, Indianapolis, IN, USA), balanced with PBS, placed in the 41 Ti Rotor (Beckman Coulter, Indianapolis, IN, USA), and centrifuged in the ultracentrifuge (UC) (Beckman Coulter Optima XPN Series, Indianapolis, IN, USA) at 167,000 x g and 4 °C for 4 h. The pellet from the previous centrifugation, containing large EVs (lEVs), was resuspended in 20 μL of PBS and transferred to a clean 1.5 mL EP (Eppendorf) tube (Eppendorf North America, Inc., Enfield, CT, USA); the falcon tube was then washed with another 10 μL of PBS to ensure as many lEVs as possible were collected. Upon completion of the centrifuge run, the supernatant was transferred to clean UltraClear ultracentrifuge tubes, balanced with PBS, placed in the 41 Ti Rotor, and centrifuged at 167,000 x g and 4 °C for 16 h. The pellet from the previous centrifugation, containing small EVs (sEVs), was resuspended in 20 μL of PBS and transferred to a clean 1.5 mL EP tube; the UC tube was then washed with another 10 μL of PBS to ensure as many sEVs as possible were collected. Upon completion of the centrifuge run, the supernatant was transferred to new Quick Seal tubes (Beckman Coulter, Indianapolis, IN, USA), balanced with supernatant, sealed, placed in the 100 Ti Rotor (Beckman Coulter, Indianapolis, IN, USA), and centrifuged at 367,000 x g and 4 °C for 16 h. The pellet from the previous centrifugation, containing exomeres (EMs), was resuspended in 30 μL of PBS and transferred to a clean 1.5 mL EP tube; the UC tube was then washed with another 20 μL of PBS to ensure as many EMs as possible were collected. Upon completion of the centrifuge run, the supernatant was transferred to new Quick Seal tubes, balanced with PBS, sealed, placed in the 100 Ti Rotor, and centrifuged at 567,000 x g and 4 °C for 16 h. The pellet from the previous centrifugation, containing supermeres (SMs), was resuspended in 30 μL of PBS and transferred to a clean 1.5 mL EP tube; the UC tube was washed with another 20 μL of PBS to ensure as many SMs as possible were collected. Upon completion of the centrifuge run, the supernatant was transferred to new Quick Seal tubes, balanced with PBS, sealed, placed in the 100 Ti Rotor, and centrifuged at 767,000 x g and 4 °C for 16 h. The pellet from the previous centrifugation, containing super-supermeres (SSMs), was resuspended in 30 μL of PBS and transferred to a clean 1.5 mL EP tube; the UC tube was washed with another 20 μL of PBS to ensure as many SSMs as possible were collected. Upon completion of the centrifuge run, the supernatant from the final ultracentrifugation was discarded. The pellet from the final ultracentrifugation, containing super-dupermeres (SDMs), was resuspended in 30 μL of PBS; the UC tube was washed with another 20 μL of PBS to ensure as many SDMs as possible were collected. All EV and nvEP samples were either immediately analyzed, stored at 4 °C and analyzed within 10 days of isolation, or stored at −80 °C and analyzed within 3 months^27^.

### 4. Size and Morphology

#### 4.a. Transmission Electron Microscopy (TEM)

Samples were prepared for TEM by applying them onto formvar/silicon monoxide-coated 200 mesh copper grids (Ted Pella Inc., Redding, CA, USA). Prior to sample application, grids were glow discharged for 60 seconds at 20 μA using a GloQube glow discharge unit (Quorum Technologies, East Sussex, UK) to improve sample adherence.

The samples were then negatively stained with NanoVan (Nanoprobes, Yaphank, NY, USA) and imaged using a Tecnai G2 Spirit TWIN transmission electron microscope (FEI, Hillsboro, OR, USA) operating at an accelerating voltage of 80 kV. Digital images were acquired with an AMT imaging system (AMT, Woburn, MA, USA).

#### 4.b. ZetaView

The sizes of lEV and sEV were analyzed *via* NTA using a ZetaView (Particle Metrix, Ammersee, Germany). The instrument was calibrated with freshly prepared standard (Alpha Nano Tech, Durham, NC, USA) at a dilution of 1:250,000 in sterile filtered Milli-Q H_2_O. Once the instrument was calibrated, the column was flushed with 2 mL of sterile filtered Milli-Q H_2_O. Each sample was diluted by a factor of 5,000 in freshly filtered Milli-Q H_2_O. A 2 mL volume of sample was added to the column, the dilution factor was set in the program, and video acquisition was initiated. It was ensured that a minimum of 8 out of the 10 cameras had suitable captures.

#### 4.c. Zetasizer

The diameters of EM, SM, SSMs, and SDMs were determined by DLS using a ZEN3600 Zetasizer Nano-ZS (Malvern Instruments Ltd., MA, United States). All measurements were performed five times at 25 °C^28^ using samples diluted in 1X PBS.

### 5. Proteomics Protocol

#### 5.a. Suspension-Trapping (S-trap^™^) processing

Unless otherwise noted all chemicals were obtained from Sigma-Aldrich. An S-trap^™^ Turbo plate (Protifi) was used for sample processing on an OT2 robot (OpenTrons). Briefly, 100 μg of each sample was reduced with 10 mM tris(2-carboxyethyl)phosphine in a heater-shaker module for 15 minutes set at 55 °C at 1,200 RPM, followed by alkylation with 20 mM iodoacetamide for 30 min in the dark at room temperature at 1,200 RPM. 27.5% phosphoric acid was used to acidify the proteins, then samples were diluted with 300 μL of S-Trap^™^ binding buffer (100 mM TEAB, 90% methanol with pH 7.1). The samples were then loaded onto S-Trap^™^ Turbo plate and washed three times with 2:1 chloroform:methanol and then three times with S-Trap^™^ binding buffer. 8 μg of Trypsin-LysC in 50 mM TEAB solution was added to the top of the S-Trap^™^ Turbo and then incubated at 37 °C for 2 h. Peptides were eluted serially with 50 mM TEAB, and 0.4% aqueous formic acid with 0.04% DDM (n-Dodecyl β-D-maltoside, GoldBio).

#### 5.b. Mass Spectrometry

Peptide samples were diluted to 0.2 μg/μL and measured by a Pierce Quantitative Fluorometric Peptide Assay (Fisher Scientific) using a Varioskan LUX multimode microplate reader (ThermoFisher). The samples (5 μL; 0.5 μg per injection) were analyzed using a Vanquish^™^ NEO liquid chromatography (UPLC) system with a μPAC^™^ Neo 50 cm column coupled to an Orbitrap Astral mass spectrometer (Thermo Fisher Scientific). Sample sets were spiked with an iRT synthetic peptide mixture, blocked and acquired in randomized order with digested E. coli injected immediately before and after every sixth sample to evaluate system performance. The consistency and reproducibility of the system performance were evaluated using QuiC (5.3.240326.57353).

#### 5.c. MS Data analysis

Raw files were searched using directDIA default settings in Spectronaut^™^ 19.5.241126.62635 F (Biognosys, Switzerland). Search data were normalized using equalized medians and log2-transformed using MSstats package (4.2.1) in R studio (R version 4.2.2). A volcano plot was based on the results of the differential abundance analysis and generated using EnhancedVolcano.

#### 5.d. Differential protein abundance and pathway enrichment analyses

Proteins identified by LC–MS/MS were quantified across the EM, SM, SSM, and SSM_EPS fractions. A protein was considered present in each fraction if it was detected in at least three biological replicates; undetected values were treated as missing. The union of all detected proteins across fractions served as the background for enrichment analyses. To characterize overall protein profiles in EM, SM, SSM, and SSM_EPS, all proteins detected in each fraction (presence sets) were submitted to IPA (QIAGEN) for Canonical Pathway and high-level functional annotation. Because this analysis was intended to characterize the functional composition of each fraction based on reproducibly detected proteins rather than identify differentially abundant proteins between fractions, no fold-change or significance threshold was applied. In parallel, WebGestalt ORA was performed using the same presence sets as input and the full quantified proteome as background^29^. ORA included Gene Ontology (GO), covering Biological Process, Cellular Component, and Molecular Function (non-redundant versions), as well as KEGG, Reactome, WikiPathways, and CORUM pathway databases. Multiple testing was controlled using the Benjamini–Hochberg false discovery rate (BH–FDR < 0.05).

Presence sets were compared to identify (i) proteins exclusive to SSM (EM–SM–SSM; three-way Venn) and (ii) proteins newly observed after EPS treatment (SSM vs SSM_EPS; two-way Venn). Venn diagrams for these comparisons were generated using the InteractiveVenn web tool^30^. As a final step, differential abundance analysis was performed using limma (version 3.64.3)^31^. IPA Core Analysis of differentially abundant proteins used thresholds of ∣log2FC∣ ≥ 1.5 and raw P-value ≤ 0.05. For volcano plots, significance was defined as ∣log2FC∣ ≥ 0.5 and BH–FDR ≤ 0.05. An in-house R-based interactive tool was used to generate volcano plots and PCA/violin visualizations.

### 6. Western Blotting

All animal procedures were conducted in accordance with the guidelines of the National Institutes of Health Guide for the Care and Use of Laboratory Animals and conformed to ARRIVE Guidelines (https://www.nc3rs.org.uk/arrive-guidelines, accessed on 9 September 2021), as approved by the Animal Care and Use Committee of the University of Nebraska Medical Centre (UNMC-IACUC Protocol #20-076-10).

#### 6.a. Sample Collection – Rat Plasma

The plasma from four male Sprague Dawley rats (130 – 210 g) was used as the positive control for the apolipoprotein A-1 and apolipoprotein B western blots in Figure 4. The plasma was collected from the sham/control rats of a previous experiment that has not yet been published. Briefly, the rats were anesthetized with 2% isoflurane delivered in 100% oxygen and placed on an electric heating pad to maintain body temperature at 37 °C throughout the experiment. Following the sham procedure, the rats were euthanized by CO_2_ inhalation. The plasma was harvested immediately after euthanizing with CO_2_ inhalation. Rat plasma was stored at −80 °C for future analysis.

#### 6.b. Sample Preparation (Protein Extraction – Cells and Rat Plasma)

Cells and rat plasma were thawed from −80 °C, incubated on ice in 100 μL of 100:1 RIPA:PIC ((50 mM Tris-HCl, 195 mM NaCl, 2 mM EDTA, 1% NP-40, 0.1% SDS):(Abcam, ab65621)) for 15 min, and then homogenized utilizing a sonicator. The soluble proteins were then extracted by centrifugation for 20 min at 13,800 x g and 4 °C. The protein concentration was measured via BSA assay and a Multiskan Sky Microplate Spectrometer (Thermo Scientific Multiskan FC, Singapore). Samples were then diluted to 2 μg/μL with RIPA. An equal volume of 2X SDS was added to each sample to further dilute the samples to 1 μg/μL and 1X SDS. The samples were then boiled in a 70 °C water bath for 2 min and immediately returned to ice^1^.

#### 6.c. Sample Preparation (Protein Extraction – EVs)

For WB analysis, EVs and nvEPs were resuspended in 100:1 RIPA:PIC in lieu of PBS after isolation by centrifugation. EVs and nvEPs were homogenized utilizing a sonicator. The protein concentration was measured via BSA assay and a Multiskan Sky Microplate Spectrometer (Thermo Scientific Multiskan FC, Singapore). Samples were then diluted to 1 μg/μL and 1X SDS using 6x SDS. The samples were then boiled in a 70 °C water bath for 2 min and immediately returned to ice^1^.

#### 6.d. Gel Electrophoresis

The protein samples (15 μg in 15 μL for each cell sample and 10 μg in varying volumes for each EVs and nvEP sample) were loaded on a precast polyacrylamide gel (NW00105BOX, Invitrogen, Carlsbad, CA, USA) along with 2 μL of Invitrogen MagicMark^™^ Western Standard and 4 μL of Bio-Rad Precision Plus Protein Dual Color Standards in separate wells. Electrophoresis was performed using a Mini Gel Tank and Blot Module Set (NW2000, Invitrogen), initially at 70 V, for approximately 20 min, and then at 120 V until the desired separation had been reached^1^.

#### 6.e. Membrane Transfer (iBlot2)

The fractionated proteins on the gel were electrically transferred onto a nitrocellulose membrane by employing Thermo Fisher Scientific iBlot2 (IB21001, Thermo Fisher Israel Ltd, Kiryat Shmona, Israel) with the preset program P0 (20 V for 1 min, then 23 V for 4 min, followed by 25 V for 2 min). The membranes were gently rinsed with Milli-Q water and then stained with Ponceau S for 5 min to visualize the total protein. Once ideal bands of the total protein were obtained, Ponceau S on the membrane was removed by rinsing it in 10 mL of 1X phosphate-buffered saline-Tween 20 solution (PBST) for 5 min^1^.

#### 6.f. Immunodetection (Antibodies)

The membranes were blocked for 30 min in 5% milk in 1X PBST at room temperature; this was followed by two 5 min washes in 10 mL of 1X PBST at room temperature. The membranes were then incubated with primary antibody in 4% BSA overnight at 4 °C. The primary antibodies used in the present experiment were purchased from Abclonal (A-) including NQO1 (A19586), GSTA2 (A7678), ApoM (A5336), ApoB (A1330), and ApoA-1 (A24290). The following day, the membranes were washed three times for 5 min in 10 mL of 1X PBST; this was followed by incubation with anti-rabbit IgG horseradish peroxidase secondary antibody (1:5000) in 5% milk in 1X PBST for 30 min at room temperature. After this, the membranes were again washed three times for 5 min in 10 mL of 1X PBST. The membranes were developed in the chemiluminescence detection reagent (SuperSignal West Dura or SuperSignal Femto; Thermo Scientific, Rockford, IL, USA). The films were scanned using the G:Box Syngene and Genesys. The intensity of the bands was quantified using the ImageJ software (version number 1.52a, NIH) and normalized for loading using Ponceau S staining^1^.

### 7. nvEP Cardioprotection of H9c2 Cardiomyocytes Against H_2_O_2_ Treatment Analyzed via CCK-8 Assay

To minimize animal use in accordance with the 3Rs (Replacement, Reduction, and Refinement), all cell cultures were maintained using Dulbecco’s Modification of Eagle’s Medium (DMEM/HIGH GLUCOSE; HyClone, Logan, UT, USA). Where animal-derived products such as fetal bovine serum (FBS; Gibco/ThermoFisher Scientific New Zealand Limited, Auckland, New Zealand) were necessary, they were sourced from certified ethical vendors complying with international animal welfare regulations. The H9c2(2-1) cell line (CRL-1446, ATCC, Manassas, VA, USA) was used in these experiments H9c2 cardiomyocytes were seeded in 96-well plates at 5,000 cells per well in 100 μL of CpM. The cells were allowed to adhere to the plate overnight. After the cells had adhered to the plate, the CpM was removed and replaced with 100 μL of CpM containing PBS (control), EM, SM, SSM, or SDMs at concentrations of 50, 100, or 200 μg/mL. The cardiomyocytes were incubated in the CpM or nvEPs at 5% CO_2_ and 37 °C for 1 h. After 1 h of nvEP treatment, 100 μL of PBS (control) or 400 μM H_2_O_2_ in CpM was added to each well. As each well already contained 100 μL of CpM with nvEPs, the final concentration of H_2_O_2_ was 200 μM. The cells were incubated in H_2_O_2_ at 5% CO_2_ and 37 °C for 3 h. After 3 h, the media was removed from each well and replaced with 100 μL of CpM and 10 μL of CCK-8. The cells were incubated with Cell Counting Kit-8 (CCK-8) at 5% CO_2_ and 37 °C for 90 min. The absorbance was measured by a Multiskan Sky Microplate Spectrometer (Thermo Scientific Multiskan FC, Singapore) at 450 nm to evaluate cell viability.

### 8. Statistical Analysis

Data are expressed as mean ± SD. For all figures, a Student’s t-test using Microsoft Excel software was used. A p value < 0.05 was considered statistically significant.

## Results

### 1. Characterization of EVs and nvEPs

While lEVs, sEVs, EM, and SM have all been characterized in previous publications^14, 17, 19^, SSMs and SDMs are novel nvEPs discovered by our laboratory.

TEM was used to visualize the morphological differences between the various subsets of the isolated EVs and nvEPs. TEM revealed that the isolated particles displayed a typical morphology with EVs showing a cup-shaped structure and lipid bilayer membrane and nvEPs exhibiting a pill-like and amembranous structure with high electron density. As seen in Figure 2 Panel A, TEM also provided a general size of the vesicles and particles (lEV: 205 nm, sEV: 126.03 nm, EM: 15.74 nm, SM: 17.76 nm, SSM: 16.20 nm, SDM: 10.79 nm) while ZetaView and ZetaSizer were used to measure the size of the vesicles and particles based on a larger sample population. As shown in Figure 2 Panel B, the average size of the lEVs is 137.68 nm while the average size of the sEVs is 116.22 nm. As ZetaView is unable to measure particle diameter below 60 nm^32^, it was exclusively used to measure the size of the EVs and dynamic light scattering (DLS) *via* a ZEN3600 Zetasizer Nano-ZS was used to measure the diameter of nvEPs (Figure 2, Panel C). The average diameters of EMs, SMs, SSMs, and SDMs were 34.94 nm, 16.18 nm, 14.10 nm, and 12.56 nm, respectively (Figure 2, Panel D).

### 2. SSMs are unique nvEPs distinct from EMs and SMs

Proteomic analysis of three of the nvEPs, EM, SM, and SSM, revealed that SSM is a unique particle as it contains 21 exclusive proteins (Figure 3 a, b, and c). Western blot analysis of one of these top proteins, apolipoprotein M (ApoM) was performed (Figure 3 d). Western blot analysis of two apolipoproteins (ApoA-1 and ApoB) was performed as a means of differentiating SSMs from HDL and LDL particles (Figure 4).

### 3. EPS of C2C12 Myotubes Evokes Upregulation of NQO1 and GSTA2 in Isolated EVs and nvEPs

Previously, our lab found that EPS upregulated antioxidant enzymes downstream of the Nrf2/antioxidant pathway, including NQO1 and GSTA2^1^ in C2C12 myotubes. Because these particles are isolated from NS- and EPS-C2C12 myotubes, we investigated whether this upregulation of NQO1 and GSTA2 was translated into their isolated particles. Western blot analysis revealed that when compared to NS controls, C2C12 myotubes that underwent EPS exhibited an upregulation of NQO1 and GSTA2. This upregulation was found in all isolated particles but to varying degrees (Figure 5).

### 4. nvEPs protect C2C12 Myotubes and H9c2 Cardiomyocytes Against H_2_O_2_-induced Injury

H9c2 cardiomyocytes were treated in one of three ways. 1) with PBS, 2) with H_2_O_2_, or 3) with an nvEP and H_2_O_2_. 1) Cardiomyocytes treated with PBS were control cells to establish 100% cell viability (light grey bar in each graph in Figure 6). 2) Cardiomyocytes treated with H_2_O_2_ were used to demonstrate H_2_O_2_-induced injury. H9c2 cardiomyocytes were treated with a range of 6.25 – 400 μM H_2_O_2_. As cell viability of the H9c2 cells treated with 200 μM H_2_O_2_ decreased by 50%, we chose this dose to test the protective effects of the nvEPs (red bars in graphs in Figure 6). 3) Cardiomyocytes were treated with nvEPs (EM, SM, SSM, or SDM) in a range of concentrations (50, 100, or 200 μg/mL of protein) for 1 h. After 1 h, 100 μL of 400 μM H_2_O_2_ was added to each well, which already contained 100 μL of CdM with nvEP or PBS, so that each well was treated with a final concentration of 200 μM H_2_O_2_ to test if nvEPs isolated from the conditioned media of non-stimulated control C2C12 myotubes (light blue bars in the graphs in Figure 6) or from the conditioned media of C2C12 myotubes that have undergone EPS (dark blue bars in the graphs in Figure 6) were cardioprotective. Conditioned media from EPS C2C12 protected H9c2 cardiomyocytes against H_2_O_2_-induced oxidative injury (Figure 6). Cardioprotection of H9c2 cardiomyocytes against H_2_O_2_ challenge was observed in cells pretreated with EM, SM, and SDM nvEPs. This cardioprotection was observed to varying degrees. Some were dose-dependent while others were not. H9c2 cardiomyocytes pretreated with EM prior to H_2_O_2_ challenge had significantly elevated levels of cell viability as compared to the control however, there was no significant difference in cell viability between EMs isolated from EPS-C2C12 or NS-C2C12. H9c2 cardiomyocytes pretreated with 100 μg/mL SM isolated from EPS-C2C12 and 200 μg/mL isolated from NS- and EPS-C2C12 prior to H_2_O_2_ challenge had significantly elevated levels of cell viability as compared to the control. However, there was no significant difference of H9c2 viability when pretreated with 50 μg/mL NS- or EPS-C2C12 or 100 μg/mL NS-C2C12 SMs. The only SM dose at which cell viability differed significantly between H9c2 cells pretreated with NS- versus EPS-derived SMs was 100 μg/mL. No significant cardioprotection against H_2_O_2_ challenge was observed in H9c2 pretreated with SSM at any dose. H9c2 cardiomyocytes pretreated with SDM showed a dose-dependent cardioprotective response against H_2_O_2_ challenge. There was no significant cardioprotection in H9c2 pretreated with 50 μg/mL SDM isolated from NS-C2C12. However, significant levels of cardioprotection were observed in H9c2 pretreated with SDM at 50 μg/mL isolated from EPS-C2C12 and 100 and 200 μg/mL isolated from NS- and EPS-C2C12. H9c2 pretreated with SDM isolated from EPS-C2C12 at concentrations of 100 and 200 μg/mL showed significantly higher levels of cell viability than those isolated from NS-C2C12 at the same concentrations.

## Discussion

A previous study in our lab demonstrated that contracting SkM activates the Nrf2-antioxidant pathway, leading to cellular adaptation against H_2_O_2_-induced toxicity. This cellular adaptation led to elevated levels of NQO1 and GSTA2, two antioxidant enzymes downstream of the Nrf2 pathway, within the SkM itself^1^. Because SkM is increasingly recognized as a secretory organ and because previous work supports a role for EV-mediated systemic communication after exercise, the present study shifted our focus from intracellular antioxidant adaptation to the potential export of antioxidant cargo in EVs and nvEPs^2, 3, 33^. Specifically, we focused on isolating EVs and nvEPs from the CdM of differentiated C2C12 myotubes after having undergone EPS and on examining the cargo that these vesicles and particles may package and potentially transport to cardiac cells.

This study has four major findings. First, serial ultracentrifugation isolated multiple particle populations from C2C12 CdM with size and morphology generally consistent with EVs and nvEPs: lEVs and sEVs showed cup-shaped morphology and lipid bilayer membranes, whereas EMs, SMs, SSMs, and SDMs showed smaller, electron-dense, amembranous or pill-like morphology. Second, EPS of C2C12 myotubes increased NQO1 and GSTA2 abundance in several isolated EV/nvEP fractions, suggesting that the contraction-induced Nrf2-antioxidant response can be reflected in extracellular particles. Third, proteomic analysis suggested that SSMs differ from EMs and SMs and contain 21 proteins not detected in those fractions. Fourth, several nvEP fractions, particularly EMs, SMs, and SDMs, protected H9c2 cardiomyocytes from H_2_O_2_-induced injury, although the magnitude and dose-dependency of protection differed between particle types and did not always track directly with antioxidant protein abundance. Together, these findings support the central hypothesis that contracting SkM can release antioxidant-enriched extracellular particles with potential cardioprotective activity.

All EVs and nvEPs were isolated using serial ultracentrifugation, as this technique is considered the gold standard for isolating particles of this size. The protocol was adapted from Zhang et al. methodology, as published in *Nature Protocols*^27^ and extended to include higher-speed and longer ultracentrifugation steps to collect smaller particles. We are confident in the technical performance of the isolation protocol employed in this study, as the size and morphological characteristics of the isolated EVs and nvEPs (lEV – SM) are consistent with previously published ranges (Figure 2)^14, 15, 17, 19, 27^. Specifically, TEM showed cup-shaped membrane-bound EVs and smaller, dense nvEP-like particles, while ZetaView and Zetasizer analyses confirmed that EVs were larger than nvEPs. However, because ultracentrifugation separates particles primarily by size, density, and sedimentation properties, it cannot fully eliminate the possibility of co-isolation of protein complexes, lipoproteins, or other non-vesicular particles. This limitation is particularly important for interpreting the smallest fractions.

We found that the same antioxidant enzymes that are upregulated in EPS-C2C12, NQO1 and GSTA2, can be packaged into the various types of EVs and nvEPs and released into the C2C12 media. Both antioxidant enzymes were significantly upregulated in lEV, sEV, EM, and SM isolated from EPS-C2C12 media as compared to NS-C2C12 media (Figure 5). This finding is important because it suggests that contraction-induced antioxidant adaptation in myotubes may not remain solely intracellular. NQO1 catalyzes the two-electron reduction of quinones and limits redox cycling, while glutathione S-transferase family members contribute to detoxification and redox buffering. Therefore, the presence of NQO1 and GSTA2 in EVs/nvEPs released from EPS-C2C12 myotubes provides a plausible mechanism by which contracting muscle could distribute antioxidant capacity to recipient cells. This concept is consistent with prior work from our laboratory proposing that SkM Nrf2 contributes to exercise-evoked systemic antioxidant defense *via* extracellular vesicular communication, as well as broader literature showing that EV cargo can influence redox status in recipient cells^24, 33, 34^.

Furthermore, we isolated what we believe to be two novel particles that have not been described previously, which we termed super-supermeres (SSM) and super-dupermeres (SDM). These particles were isolated by building on Zhang et al.’s methodology^27^ by further extending the duration and speed of the conditioned media centrifugation (Figure 1). Their morphology, specifically their amembranous and pill-like shape, aligns with previously described nvEPs, supporting the possibility that these are novel nvEP-like particles^16^. These novel particles are distinguished from known nvEPs by both size and cargo. SSMs and SDMs are smaller (14.10 nm and 12.56 nm, respectively) than EMs and SMs, which typically range from 28 – 50 nm and 15 – 25 nm^35^, respectively (Figure 2). Proteomic and bioinformatic analyses provide further evidence that SSMs are unique particles, as they contain 21 proteins not found in EMs or SMs (Figure 3 A, B, C). Western blot analysis comparing the protein expression of apolipoprotein M (ApoM) among EMs, SMs, and SSMs further supported the proteomic findings because ApoM expression was higher in the SSM fraction (Figure 3, D).

At the same time, the ApoM finding raises an important interpretive question. ApoM is an apolipoprotein that is primarily bound to high-density lipoproteins (HDLs) in the plasma^36^, and HDL particle diameters overlap with the measured sizes of SSMs and SDMs. Thus, these fractions could represent novel nvEP-like particles, HDL, or HDL-related particles, or a mixed population containing both. This question cannot be resolved by size and morphology alone. HDL particle sizes range from 5 to 17 nm and are broken up into subclasses based on their size and cellular responsibilities. These subclasses are defined as follows: 7.8, 8.4, 9.6, 12.2, and 17.0 nm in diameter. HDL particles are also isolated via density-gradient ultracentrifugation however; their isolation is achieved by ultracentrifugation at lower speeds (65,000 rpm) and shorter runs (3 h) than those used to isolate the SSMs (567,000 x g for 16 h). As these SSMs fall within the size range of HDLs and proteomic analysis revealed that SSMs contain a lipoprotein, we would be remiss not to check these SSMs for the HDL protein marker, apolipoprotein A-1 (apoA-1). Figure 4 shows that apoA-1 protein is present within the ultracentrifuge pellets of all four subgroups of nvEPs but is significantly higher in SSMs and SDMs, suggesting these particles may be a subgroup of HDLs and not novel nvEPs. To address the question of whether these particles could be lipoproteins instead of nvEPs, we also checked each subgroup of nvEPs for apolipoprotein B (ApoB) as one ApoB molecule is attached to all atherogenic particles (LDL, VLDL, and IDL)^37^. Figure 4 shows that ApoB was found at lower intensity in SSMs and SDMs compared with EMs and SMs; therefore, we can confidently say that our two new particle fractions, SSMs and SDMs, are no more likely to be atherogenic particles than previously published EMs and SMs. With the findings from Figures 2 - 4, we cannot yet verify whether these particles are in fact new nvEPs or whether we have discovered a new cardioprotective role of HDL particles^38^.

These findings reveal a major limitation of this study and its use of gradient ultracentrifugation. Ultracentrifugation enriches particles based on sedimentation behavior but does not prove that each pellet contains a single particle class. While this method of isolation is considered the gold standard within the field of EVs and nvEPs, it requires careful characterization and complementary validation because samples may include EVs, nvEPs, lipoproteins, protein aggregates, or other co-isolated macromolecular structures^39, 40^. Therefore, future studies should combine ultracentrifugation with orthogonal approaches such as density gradient separation, size-exclusion chromatography, immunocapture, or AF4, and should test established positive and negative markers for EVs, nvEPs, and lipoproteins. These steps would strengthen confidence that antioxidant cargo and protective activity are carried by the intended particle populations.

Although NQO1 and GSTA2 are present in lEV – SSM particles, the degree of upregulation differed between particles isolated from NS- vs EPS-C2C12 myotubes. Similarly, these particles offered differing levels of protection of H9c2 cardiomyocytes against H_2_O_2_-induced injury. Three of the four types of nvEPs tested, EM, SM, and SDM, showed cardioprotective effects against H_2_O_2_-induced injury, whereas SSM did not show cardioprotection against H_2_O_2_ challenge (Figure 6). However, the dose at which protection was observed and the level of protection differed between particle types. EMs showed protection at all three particle concentrations (50, 100, and 200 μg/mL). However, there was no significant difference in cardioprotection observed between EMs isolated from NS/control-myotubes and those isolated from EPS-myotubes. SMs showed cardioprotection at all three concentrations (50, 100, and 200 μg/mL). SMs showed significantly different levels of cardioprotection between SMs isolated from NS/control-myotubes and those isolated from EPS-myotubes at 100 μg/mL but no significant difference in cardioprotective levels between NS/control-myotubes and EPS-myotubes at 50 or 200 μg/mL. SDMs showed a dose-dependent cardioprotective response, with no protection at 50 μg/mL but protection at 100 and 200 μg/mL.

The variability of cardioprotection observed between particle types was not as expected. We anticipated that if there was to be a difference in cardioprotection, it could be attributed to cargo within the particles, i.e., we thought that the larger particles would show greater cardioprotection as they would be able to hold more antioxidant enzymes. We also anticipated that the particles isolated from EPS-treated myotubes would induce greater cardioprotection as they contain a greater concentration of antioxidant enzymes. The results only partially supported this expectation. The reasoning for the variability observed in cardioprotection between doses of particles and between particle types remains unclear and should be further investigated. One possible explanation is that total antioxidant enzyme abundance by Western blot does not necessarily reflect the bioavailable or functional antioxidant activity delivered into H9c2 cells. Another possibility is that uptake efficiency differs between particle populations, so a particle with less antioxidant cargo could still have a stronger biological effect if it is taken up more efficiently. A third possibility is that the protective effect depends on multiple cargo classes, including proteins, lipids, and nucleic acids, rather than NQO1 and GSTA2 alone. Finally, high concentrations of some particles may have complex or even non-beneficial effects, which could explain why protection was not uniformly dose dependent. These possibilities should be further investigated by measuring particle uptake, intracellular ROS, apoptosis/necrosis markers, mitochondrial function, and direct antioxidant activity after treatment.

The cardioprotection observed with pretreatment of the AOE-nvEPs provides researchers with a potential mechanism by which exercise and remote preconditioning in animals and humans may induce cardioprotection^41-43^. This finding is consistent with previous reports that exercise-induced circulating EVs protect against cardiac I/R injury and that remote ischemic conditioning can transfer cardioprotection through plasma EVs^4, 44, 45^. The present study extends this concept by focusing specifically on contracting SkM as a potential source of antioxidant-enriched EVs/nvEPs and by demonstrating protection in H9c2 cells against H_2_O_2_-induced oxidative injury.

## Conclusion

In summary, this study identified two new nvEPs which are smaller in size and possess distinct protein profiles compared to previously described particles. The study also demonstrated that EVs/nvEPs isolated from the CdM of EPS-C2C12 myotubes contain increased NQO1 and GSTA2, two antioxidant enzymes downstream of the Nrf2/antioxidant pathway. Functionally, selected nvEP fractions protected H9c2 cardiomyocytes against H_2_O_2_-induced oxidative injury, supporting a potential molecular mechanism by which contracting SkM may protect cardiac cells through transfer of antioxidant-enriched extracellular particles. Further studies are needed to define the identity of the novel particle fractions and distinguish nvEPs from lipoprotein-related particles.

## Figures and Tables

**Figure 1.**
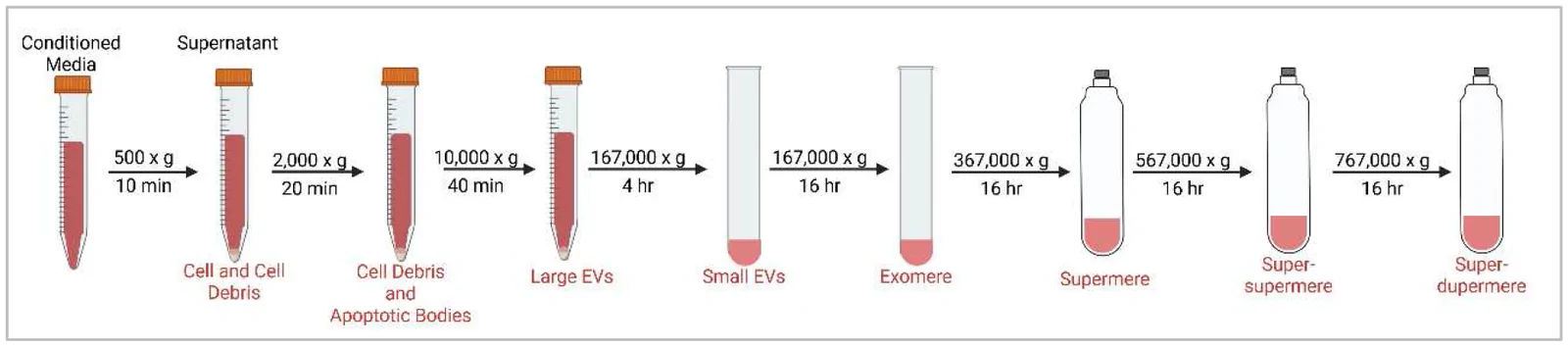
EV and nvEP Isolation Protocol. Graphical representation of the EV and nvEP isolation protocol as described below.

**Figure 2.**
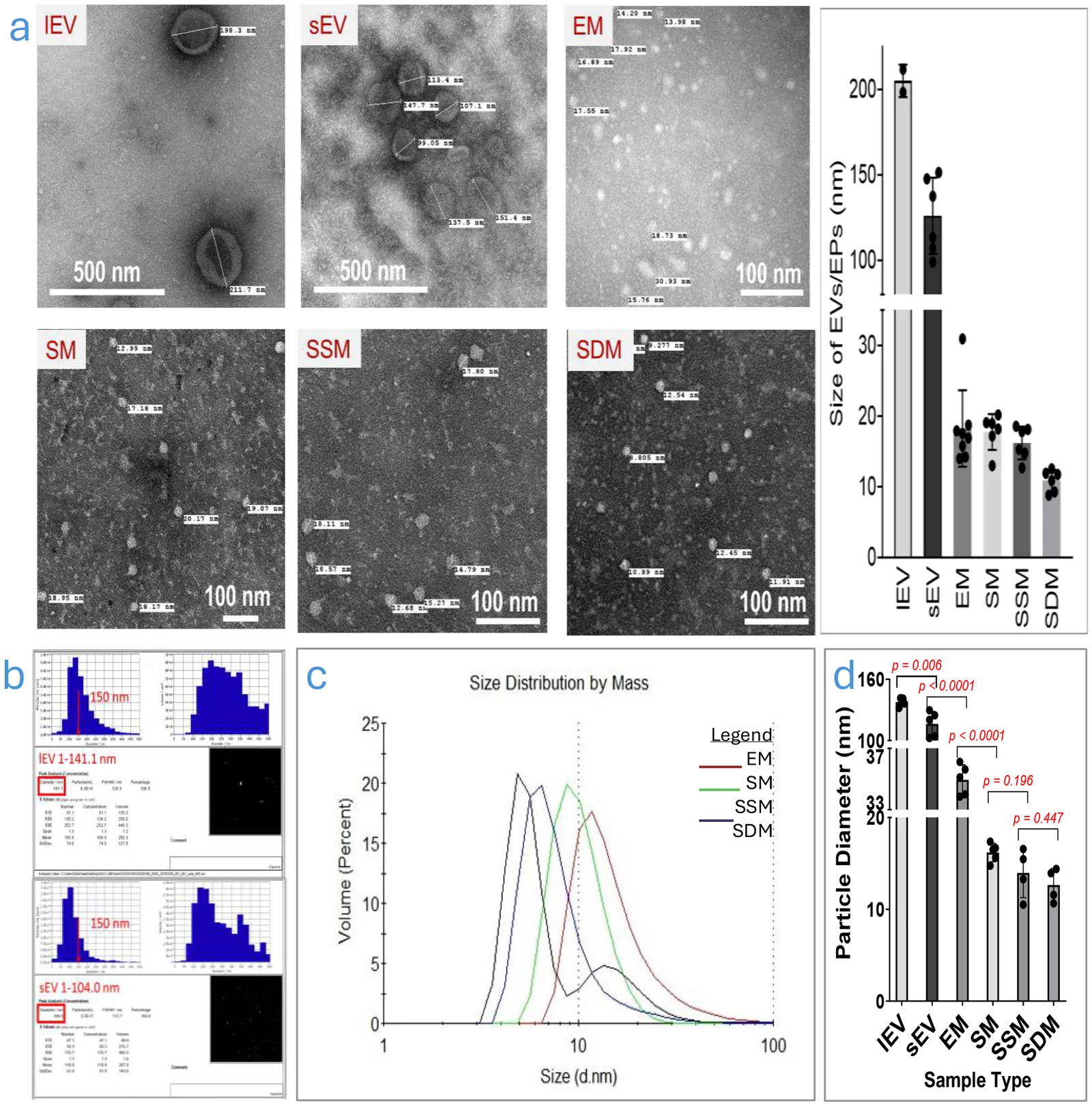
Size and Morphology of EVs and nvEPs. Size and morphology of extracellular vesicles – large (lEV) and small (sEV) and non-vesicular extracellular nanoparticles (nvEPs) – exomeres (EM), supermeres (SM), super-supermeres (SSM), and super-dupermeres (SDM), determined by a) Transmission Electron Microscopy (TEM), b) ZetaView, and c) Zetasizer. Representative tracings of EM, SM, SSM, and SDM size distribution by mass from. d) Average vesicle and particle diameter in nanometers (nm), calculated from ZetaView or Zetasizer data.

**Figure 3.**
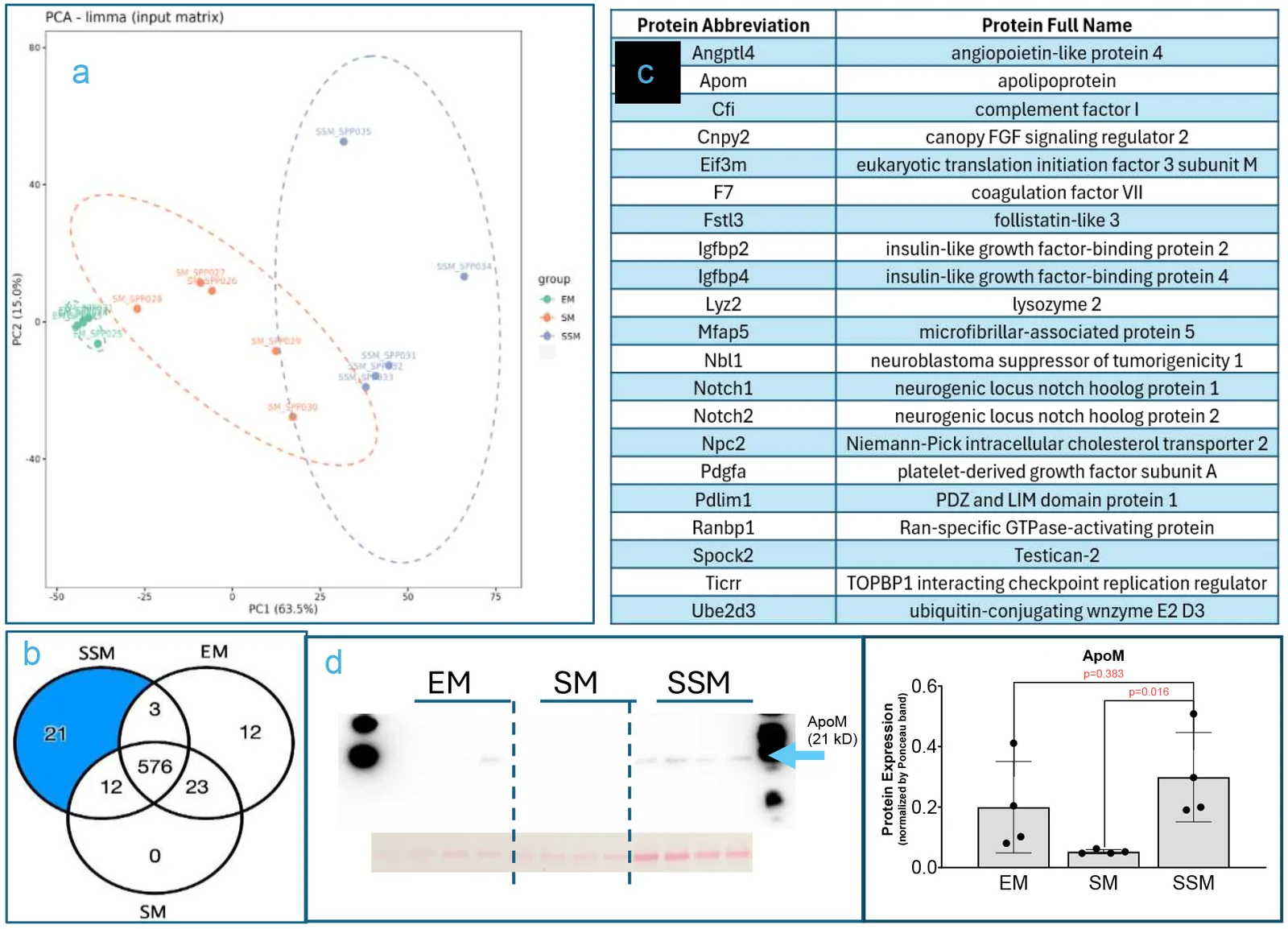
Identifying SSMs as a Unique nvEP. a) Principal component analysis (PCA) plot of log2-intensity profiles from EM, SM, and SSM runs. Each point is one run; colors denote fractions. Axes show variance explained by PC1 and PC2. Dashed ellipses visualize group dispersion. b) Venn Diagram showing 21 proteins unique to SSM particles. c) List of 21 proteins unique to SSM (listed alphabetically). d) Protein expression of ApoM is significantly higher in SSMs as compared to SMs.

**Figure 4.**
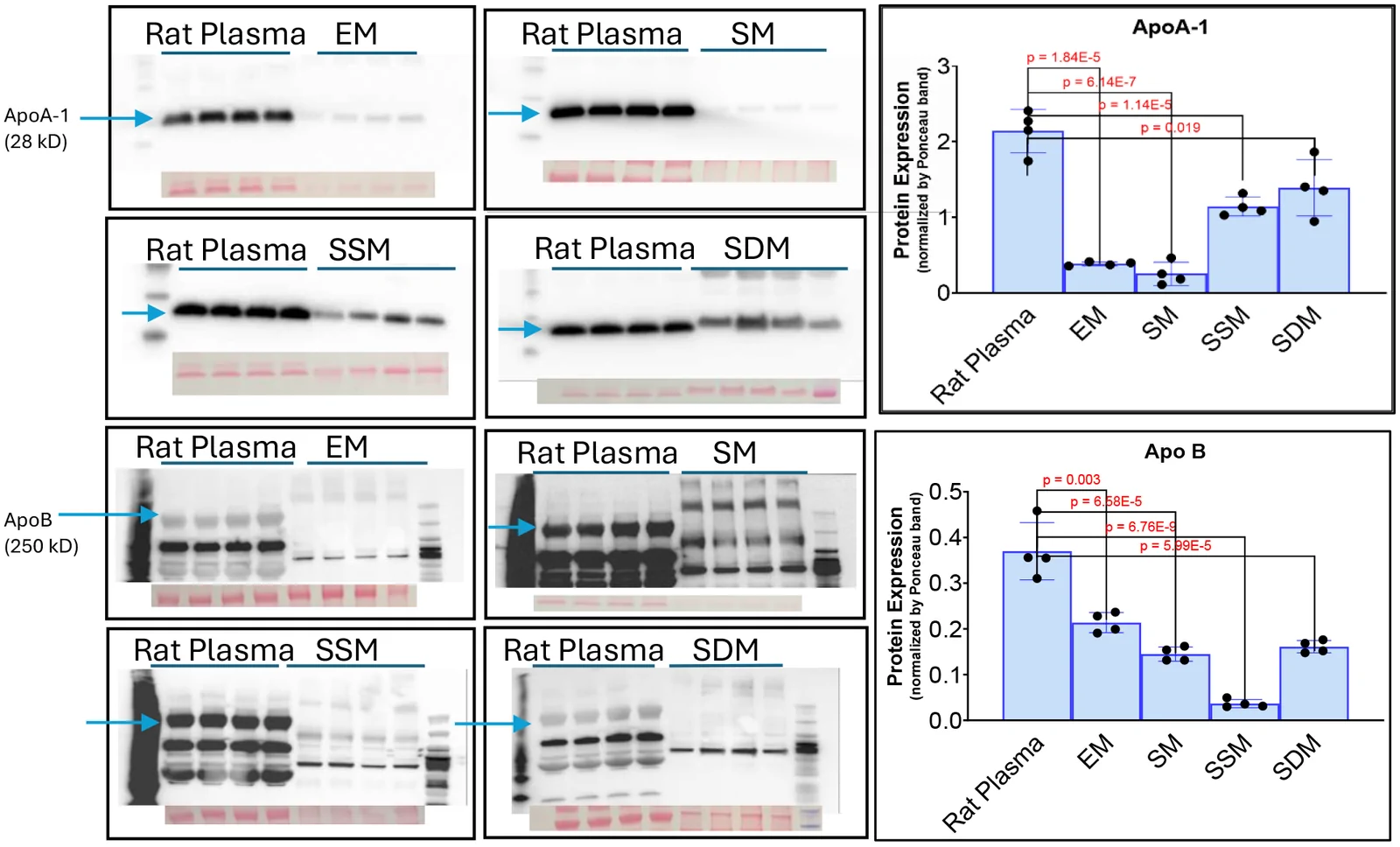
ApoA-1 and ApoB Protein Expression. Protein expression of ApoA-1 (top) and ApoB (bottom) in all four nvEPs. Rat plasma was used as the positive control. P-values calculated using Student’s T-test. n =4/group

**Figure 5.**
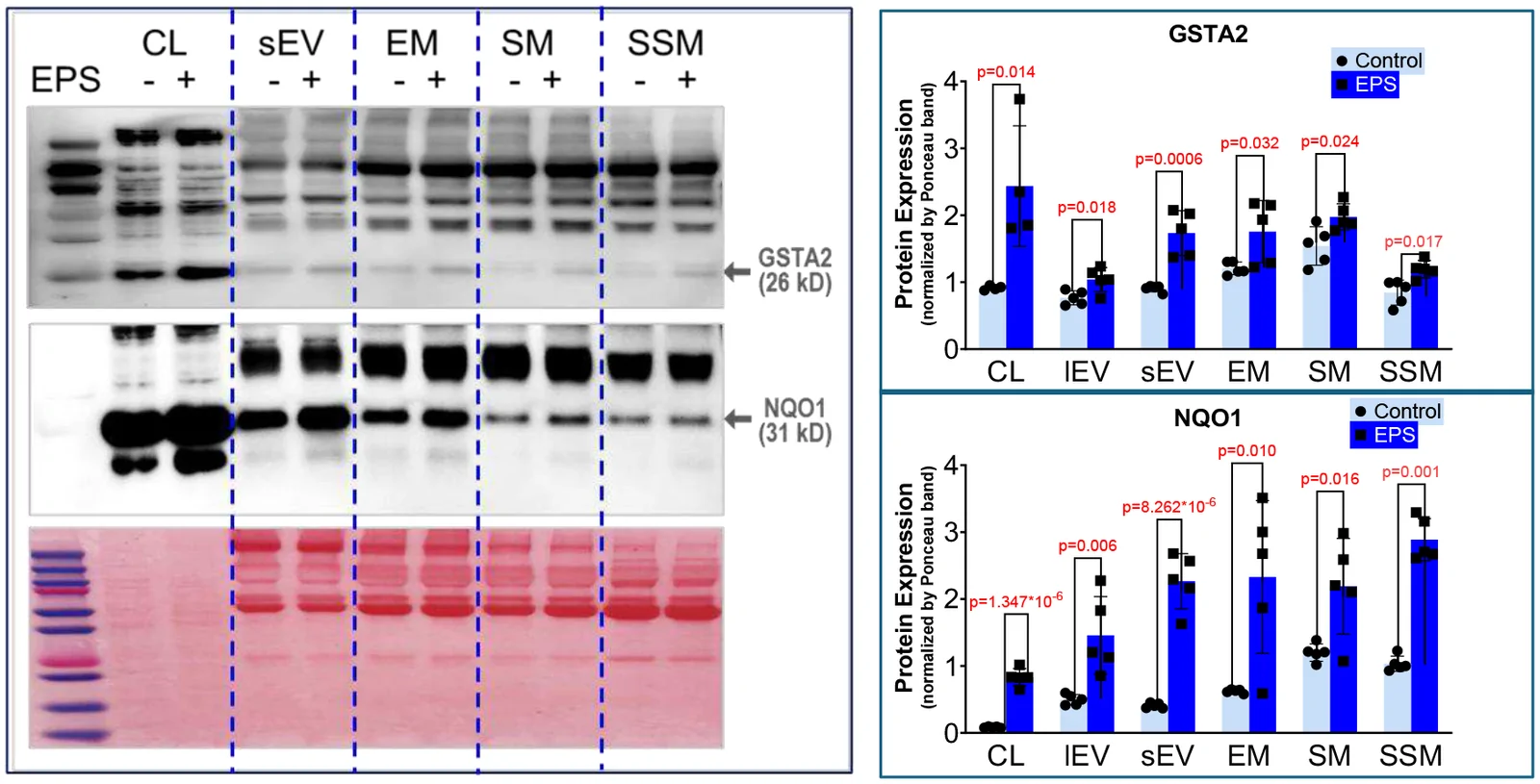
GSTA2 and NQO1 Western Blots. Western blot depicting protein expression of glutathione S-transferase alpha 2 (GSTA2) and NAD(P)H quinone oxidoreductase 1 (NQO1) of extracellular vesicles (EVs) – large (lEV) and small (sEV) and non-vesicular extracellular nanoparticles (nvEPs) – exomeres (EM), supermeres (SM), and super-supermeres (SSM), isolated from C2C12 myotubes that either underwent electrical pulse stimulation (EPS +) (dark blue bars) or did not and served as control (EPS −) (light blue bars). P-values calculated using Student’s T-test. n = 4 – 5/group

**Figure 6.**
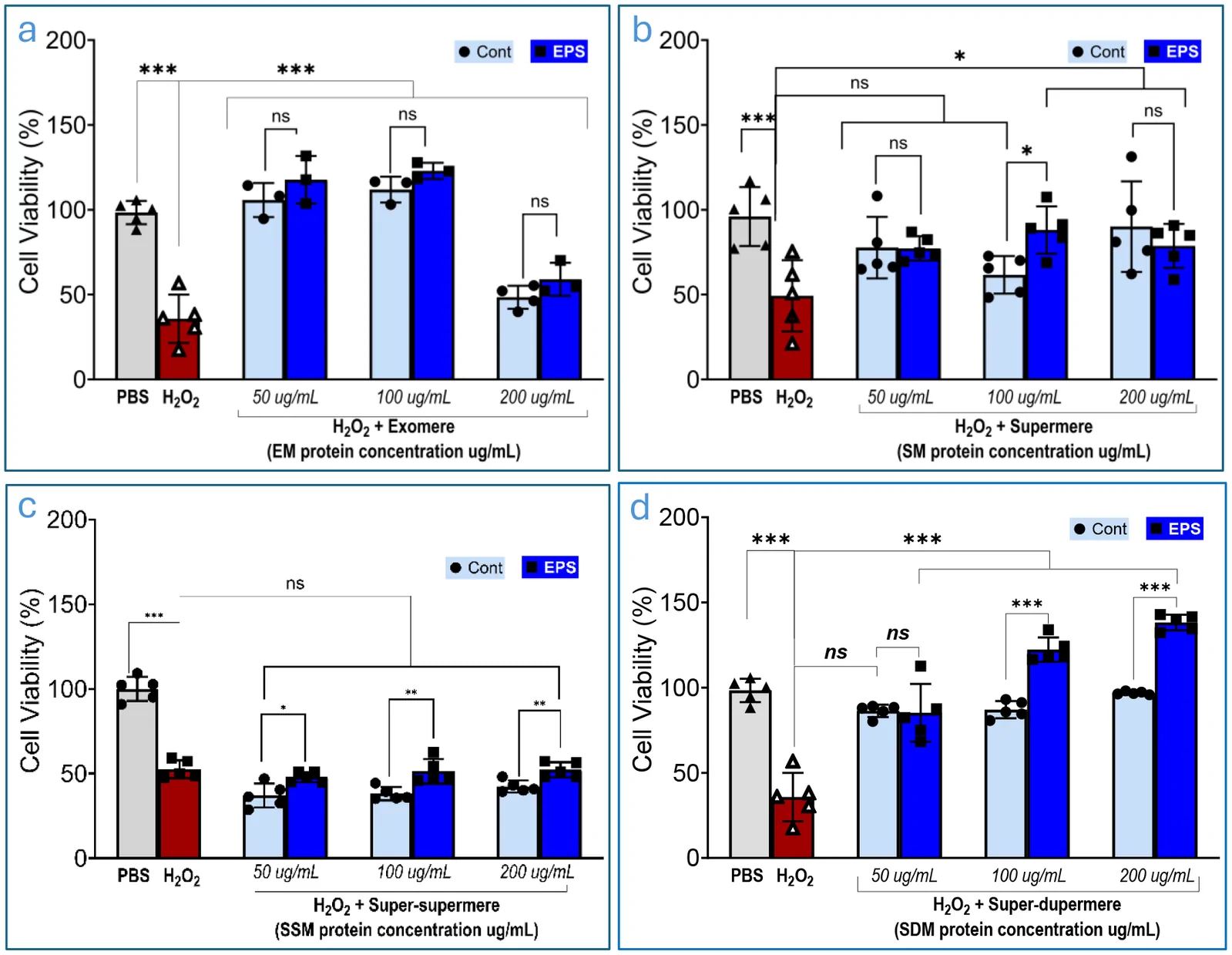
Cardio protection of nvEPs Against Oxidative Stress. Differences in cell viability of H9c2 cardiomyocytes against 200 μM H_2_O_2_-induced injury when pretreated with non-vesicular extracellular nanoparticles (nvEPs) – a) exomeres (EM), b) supermeres (SM), c) super-supermeres (SSM), and d) super-dupermeres (SDM). Gray bars = PBS/Veh Treated. Red bars = H_2_O_2_ only. Light blue bars = EPS−. Dark blue bars = EPS+. P-values calculated using Student’s T-test. *p < 0.05; **p < 0.01; ***p < 0.001.

## Data Availability

The mass spectrometry proteomics data generated in this study have been deposited to the ProteomeXchange Consortium via the PRIDE partner repository with the dataset identifier PXD084099. The R scripts used for downstream bioinformatics analyses and visualization are available from the corresponding author upon request. All other original contributions presented in this study are included in the article. Further inquiries can be directed to the corresponding authors.
